# Effect modification of *FADS2* polymorphisms on the association between breastfeeding and intelligence: protocol for a collaborative meta-analysis

**DOI:** 10.1136/bmjopen-2015-010067

**Published:** 2016-08-01

**Authors:** Fernando Pires Hartwig, Neil Martin Davies, Bernardo Lessa Horta, Cesar Gomes Victora, George Davey Smith

**Affiliations:** 1 Postgraduate Program in Epidemiology, Federal University of Pelotas, Pelotas, Brazil; 2 MRC Integrative Epidemiology Unit, University of Bristol, Bristol, UK

**Keywords:** Breast Feeding, Intelligence, FADS2, Docosahexaenoic Acids, Meta-analysis

## Abstract

**Introduction:**

Evidence from observational studies and randomised controlled trials suggests that breastfeeding is positively associated with IQ, possibly because breast milk is a source of long-chain polyunsaturated fatty acids. Different studies have detected gene-breastfeeding interactions involving *FADS2* variants and intelligence. However, findings are inconsistent regarding the direction of such effect modification.

**Methods/design:**

To clarify how *FADS2* and breastfeeding interact in their association with IQ, we are conducting a consortium-based meta-analysis of independent studies. Results produced by each individual study using standardised analysis scripts and harmonised data will be used. *Inclusion criteria*: breastfeeding, IQ and either rs174575 or rs1535 polymorphisms available; and being of European ancestry. *Exclusion criteria*: twin studies; only poorly imputed genetic data available; or unavailability of proper ethics approval. Studies will be invited based on being known to have at least some of the required data, or suggested by participating studies as potentially eligible. This inclusive approach will favour achieving a larger sample size and be less prone to publication bias.

**Discussion:**

Improving current understanding of *FADS2*-breastfeeding interaction may provide important biological insights regarding the importance of long-chain polyunsaturated fatty acids for the breastfeeding-IQ association. This meta-analysis will help to improve such knowledge by replicating earlier studies, conducting additional analysis and evaluating different sources of heterogeneity. Publishing this protocol will minimise the possibility of bias due to post hoc changes to the analysis protocol.

**Strengths and limitations of this study**

- Standardised statistical analysis of harmonised data will improve comparability between studies.
- Attempts to include both published and unpublished studies will minimise the possibility of publications bias.
- It will not be possible to fully harmonise exposure and outcomes measures.
- Additional sources of heterogeneity will likely remain.
- Elaborating and reporting the analytical plan before data analysis will protect against biased reporting.

## Introduction

Consortium-based efforts have been proposed as a practice that may contribute to generate more reliable scientific findings.^1^ Such an approach has many desirable characteristics, including improved power by increasing sample size, harmonisation of variables and analyses, and avoiding winner's curse bias. Adapting a similar approach used in a previous work on 5-HTTLPR, stress and depression,^2^ this manuscript describes the protocol for a collaborative meta-analysis on the interaction between breastfeeding and *FADS2* polymorphisms when IQ is the outcome. As described previously,^2^ publishing the protocol is important for several reasons. These include: avoiding biased reporting by documenting study protocol and design, as well as primary analysis, prior to conducting and publishing the study; facilitate the understanding of the results of the study by its readership when it is completed; and help similar initiatives in the future to elaborate a protocol and encourage this practice as a means to improve transparency and commitment to the analysis plan defined a priori.

## Background

There is substantial evidence of short-term health benefits of breastfeeding by reducing children morbidity and mortality from infectious diseases.^3^ ^4^ Basing on this evidence, WHO^5^ and the United Nations Children's Fund^6^ recommend that every child should be exclusively breastfed for 6 months, with partial breastfeeding continued until 2 years. More recently, associations between breastfeeding and positive health outcomes in adulthood suggest that breastfeeding might also have long-term effects.^4^ ^7–9^

Different epidemiological studies have detected positive associations between breastfeeding and intelligence-related outcomes.^7^ ^9^ Residual confounding has been suggested to influence much of the findings involving breastfeeding and child cognitive development.^10^ However, randomised trials provided evidence that breastfeeding causes increased motor development during the first year of life,^11^ as well as intelligence measured in healthy infants participating in the Promotion of Breastfeeding Intervention trial.^12^ Additional evidence of health benefits of breastfeeding from randomised studies includes better cardiovascular risk profile (lipoprotein profile^13^ and blood pressure^14^) in preterm-born children at 13–16 years. Long-term observational associations with IQ have also been detected. For example, a recent population-based study in South Brazil (where breastfeeding is not associated with socioeconomic position at birth) identified a positive association with IQ in individuals aged 30–31 years; this association captured 72% of the association of breastfeeding with income.^15^ This raises the possibility that breastfeeding not only influences health, and also intellectual human capital and economic productivity.^15^

Given the nature of the interventions in some of the aforementioned trials,^13^ ^14^ at least some of the effects of breastfeeding are hypothesised to be biological. Regarding intelligence, a potential mechanism is that breast milk is a source of long-chain polyunsaturared fatty acids (LC-PUFAs) including docosahexaenoic acid (DHA), which have been implicated in brain development.^16^ ^17^ It has been hypothesised that the association between breastfeeding and IQ could differ according to the capacity to synthetise DHA from metabolic precursors.^18^ Special attention has been given to genetic variation in the *FADS2* gene, which encodes a protein involved in desaturation processes required for endogenous synthesis of LC-PUFAS from shorter chain fatty acids.^19^ ^20^

Caspi and colleagues provided evidence for *FADS2*-breastfeeding interaction involving two *FADS2* variants: rs174575 (major/minor allele: C/G) and 1535 (major/minor allele: A/G). In two independent samples, breastfeeding was positively associated with IQ in non-G carriers, but not in GG individuals.^18^ However, these results were not in accordance with the DHA hypothesis, since rs174575 -G allele has been associated with lower LC-PUFAs levels in serum^21^ and plasma^22^ in large studies, although smaller (and possibly underpowered) studies failed to detect such associations.^23^ ^24^ Therefore, GG individuals would be expected to benefit more from breastfeeding than their counterparts. Indeed, a subsequent *FADS2*-breastfeeding interaction study using a larger sample obtained results consistent with this hypothesis, with the strongest association occurring in GG individuals.^20^ On the other hand, three twin studies failed to detect any interaction.^25–27^ One of them failed to demonstrate a dose-response trend.^25^ Another study observed a negative trend between breastfeeding and IQ at age 18 years, but CIs were large, and the same trend was not observed for educational attainment at age 12 years.^26^

There may be several heterogeneity sources that will be discussed below:

1. *Study design*: Several design aspects can influence results. One such aspect is sample size, and publication bias is due to the selective publication of small studies with positive results. Sample size is particularly important for this meta-analysis because the ongoing debate relates to the association of breastfeeding and IQ among GG individuals (minor allele homozygotes), which prevalence is expected to be ∼12.9% (rs1535) and 7.2% (rs174575) in European ancestry samples based on estimates from the 1000 Genomes Project (phase 3). Another issue is that several of the published studies collected breastfeeding information retrospectively at different offspring ages (2 years,^26^ 2–3 years,^18^ 10 years,^27^ 12 or 16 years,^25^ and 5–33 years^26^), while one study used prospective data.^20^ Retrospective measurements might be subjected to recall bias. We will evaluate the role of study design characteristics as sources of heterogeneity.
2. *Sample characteristics*: General sample characteristics may influence the results due to non-modelled interactions or different confounding structures. For example, a cross-cohort comparison evidenced that the association between breastfeeding and socioeconomic position is different between the British Avon Longitudinal Study of Parents and Children (from a high-income population) and the Brazilian 1993 Pelotas Birth Cohort (from a middle-income population).^28^ Another important aspect is ethnicity because genetic epidemiology studies in multiethnic samples are subjected to bias from population stratification.^29^ Moreover, samples from different ethnicities may differ regarding underlying linkage disequilibrium structure. In case of indirect association, this could introduce heterogeneity due to differential associations between the genotyped variant(s) with the causal variant(s) between ethnicities.^30^ Another point related to both sample characteristics and study design is twin studies. Systematic differences in breastfeeding have been observed when comparing singletons and twins,^31^ ^32^ which could limit the comparability of results. We therefore opted by limiting the meta-analysis to singletons of European ancestry. We will also investigate the contribution of other sample characteristics to between-study heterogeneity.
3. *Limited breastfeeding information*: In addition to breastfeeding prevalence, other factors such as duration and quality (eg, exclusive vs non-exclusive) are important when studying the association of breastfeeding with any outcome of interest. Owing to all *FADS2*-breastfeeding interaction studies published so far having used breastfeeding as a binary (never vs ever breastfed), important information is likely being lost. For example, it is not possible to do a fair comparison using a binary breastfeeding variable when the samples greatly differ regarding average breastfeeding duration. On the other hand, using three or more categories of breastfeeding may incur in power issues when evaluating interactions. Therefore, we will use (whenever available) more detailed breastfeeding data to gain insights such as whether there is a dose-response pattern given that power issues are likely to be reduced. We will also evaluate whether breastfeeding characteristics (eg, prevalence and duration) contribute to heterogeneity.
4. *Timing and nature of IQ measurements*: The aforementioned studies measured IQ using different tests or comprising different subtests and at different ages. These are potential sources of heterogeneity, which will be explored in our analysis. To improve numerical comparability across studies, IQ measurements will be converted to sample-specific Z-scores prior to analysis.

### Study objectives

The general aim of our study is to contribute to clarify how *FADS2* variants and breastfeeding interact regarding their association with IQ. We will address this research question by conducting a collaborative meta-analysis using results from de novo standardised analyses performed by collaborators using variables determined before data analysis.

Our study will test the following main hypotheses:

1. The association between breastfeeding and IQ is different among GG individuals compared to non-G carriers.
2. Using more detailed breastfeeding data rather than a dichotomous variable will provide additional insights (eg, whether or not a dose-response relationship exists).
3. Factors associated with study design or sample characteristics are sources of between-study heterogeneity.

It is possible that a posteriori hypotheses based on exploratory analysis emerge. In case they occur, they will be clearly indicated as such when reporting results.

## Methods/design

### Overview

The coordinating team defined the analytical plan, inclusion criteria and variables to be analysed a priori. The overall guideline for such definition was to properly replicate previous investigations based on a binary variable for breastfeeding (eg,^18^ and^20^), as well as including additional analyses (eg, evaluation of dose–response), while adjusting for important potential confounders.

As previously described,^2^ using de novo results in a collaborative meta-analysis has several desirable aspects. These include analysis of harmonised data using consistent analytical approaches (such as statistical tests and covariate adjustment), inclusion of unpublished data and possibility of performing secondary analysis. Statistical analysis of each individual study will be performed by its own investigators using standardised scripts developed by the coordinating team. A detailed analysis plan describing how to use the scripts provided, and how they work, will be distributed to the analysts.

### Eligibility criteria

Studies will be considered eligible for this study if they meet all the following criteria:

1. Data availability. The minimal data required for eligibility is:
  - Binary (never vs ever) breastfeeding variable (either any or exclusive breastfeeding);
  - IQ measured using standard tests;
  - At least one of the two *FADS2* polymorphisms considered: rs174575 and rs1535—both genotyped and imputed will be included.
2. Ancestry. To avoid population stratification and ancestry effects, only samples of European ancestry are eligible. Multiethnic studies will be eligible if they can identify a subsample of European ancestry. Whenever possible, such classification will be based on ancestry-informative principal components (see ‘Study variables’ for details), although other indicators (eg, self-reported skin colour) will also be considered.
3. Study design. Prospective and retrospective cohort studies will be included.

Exclusion criteria for this study are:

1. Genetic data. The only genetic data available is imputed, and its imputation quality (eg, r² and INFO metrics of MACH and IMPUTE, respectively^33^) is below 0.3.
2. Study design. Twin studies will not be included.
3. Ethical issues. Studies that do not have appropriate ethical approval to use their data as this study requires will be excluded.

### Identifying studies

Our aim is to invite all eligible studies to participate, regardless of having published or not on this topic. Doing so will favour the achieving of a larger sample size and minimise publication bias. Invitations will be sent to groups that are known by the coordinating team to have at least some of the data required available, and suggested by participating groups as possibly eligible. Although this approach is likely unspecific (ie, we expect that some of the contacted studies are not eligible), it is useful for improving sensitivity.

Following an initial contact, the analysis plan will be distributed to studies interested in participating. This has two main goals: identify eligible studies and obtain feedback regarding the analysis plan. One or more individual studies will be invited to run preliminary analysis using the code developed by the coordinating team in order to identify and correct potential issues before distributing the code to all contributing studies.

### Study variables

1. Breastfeeding. The simplest form will be as a binary variable (never vs ever breastfed). Whenever breastfeeding duration is available, four additional breastfeeding variables will be considered: binary (<6 months and ≥6 months) categorical (none, >0 and ≤1, >1 and ≤3, >3 and ≤6 months and >6 months), numerically coded categorical (for linear trend tests) or numeric (in months) variable. For studies with information regarding breastfeeding quality (ie, any vs exclusive), all breastfeeding variables will be generated twice, corresponding to each quality category.
2. IQ. Different IQ measures that yield an approximately normally distributed numerical variable will be included. To improve numerical comparability, such measures will be converted to sample Z-scores (ie, for each observation, subtract the mean and divide by the SD). However, this does not imply in comparability regarding other aspects, such as type of test or subtests included. Since limiting based on such aspects would be too restrictive, we opted by being less stringent in this regard. The influence of such differences will be evaluated at the meta-analysis stage.
3. FADS2 polymorphisms. We will use two single nucleotide polymorphisms (SNPs) in the *FADS2* gene: rs174575 and rs1535. Each SNP is a three-level variable, depending on how many copies an individual carries of the rarest (G) allele. The levels are: no copies of the G allele (ie, two copies of the major allele); one copy of the G allele and one copy of the major allele (heterozygous), and two copies of the G allele (ie, homozygous G). The genotypes corresponding to each of these levels are CC, CG and GG for rs174575; and AA, AG and GG for rs1535. G is expected to be the rarest allele in Europeans samples, with a frequency of about 25.5% and 35.0% for rs174575 and rs1535, respectively. Importantly, since C pairs with G, strand-orientation issues related to the rs174575 variant can only be detected by comparing observed with expected allele frequencies. As a quality control check, the analysis script will stop if the G-allele frequency is outside the range of 10–40%. Both genotyped and imputed SNPs will be considered. If imputed, dosages corresponding to the G allele rather than ‘best-guess’ genotypes will be used. Each polymorphism will be coded in four different forms, reflecting distinct genetic effects: additive or per-allele, corresponding to the number of copies of the G allele (AA or CC=0, AG or CG=1, GG=2); dominant, where G-allele carriers are compared to non-G-carriers (AA or CC=0, AG/GG or CG/GG=1); recessive, where GG individuals are compared with A-allele or C-allele carriers (AA/AG or CC/CG=0, GG=1); and overdominant, where heterozygous are compared with homozygous individuals (AA/GG or CC/GG=0, AG or CG=1).
4. Covariates. This study will include the following covariates:
  - Sex (male/female).
  - Age at IQ measurement (in years) and age^2^ (to account for potential non-linear age effects).
  - Ancestry-informative principal components^34^ for studies with genome-wide genotyping data available. Such components (calculated within the European subsample using a subset of independent SNPs of minor allele frequency >1%) will be used to account for residual population stratification.
  - Measures of maternal education or maternal cognition. To achieve international comparability, maternal education will be coded according to the 1997 International Standard Classification of Education (ISCED) of the United Nations Educational, Scientific and Cultural Organization.^35^ To improve numerical comparability across studies (relevant to sensitivity analysis), maternal cognition will be converted to sample Z-scores. In studies that measured these variables more than once (ie, at different time points), the closest time point to offspring birth will be used. Adjusting for these variables will be performed similarly to age at IQ measurement to account for potential non-linear effects.
  - A categorical indicator of field centre for multicentric studies. This will be used to account for eventual batch effects.
  - Any other recommended study-specific indicators, if considered necessary by the coordinating team.

### Statistical analysis

1. *Overview and preanalysis steps*: The scripts were written in R (http://www.r-project.org) due to its free availability and widespread use. Two scripts were produced. One is called ‘user's script’ and is aimed at being used by the analysts. It contains <200 lines of code, and the vast majority are comment lines explaining how to conduct each step with examples. The other is called ‘developer's script’, which contains the actual functions that will perform quality control checks, calculate summary statistics and perform association analysis in >1000 lines of code. By providing a simplified script that uses more complicated functions from an accompanying script, we hope to reduce the work burden of contributing studies.
  To ensure consistency across studies, only the coordinating team will make any eventual modifications in the developer's script. So, in case an analyst identifies an issue, it will be reported to the coordinating team who will make any revisions if necessary, and redistribute the code.
  The main task of the analysts will be to format the data for the analysis. The analysis plan will contain detailed instructions on how the data should be formatted. To minimise harmonisation issues, the first step of the analysis will be a series of quality control checks regarding general data formatting, eligibility criteria, categorical variable levels, outliers (defined as being outside the range of ±4 SDs from the mean) and impossible numbers (eg, negative IQ points) in continuous variables. After the quality control step, summary statistics for the sample and for the SNPs will be generated. These will be used at the meta-analysis stage to identify potential heterogeneity sources.
2. *Association analysis*: Association analysis will be performed by linear regression with heteroskedasticity robust SEs. The main statistical model underlying all analysis is
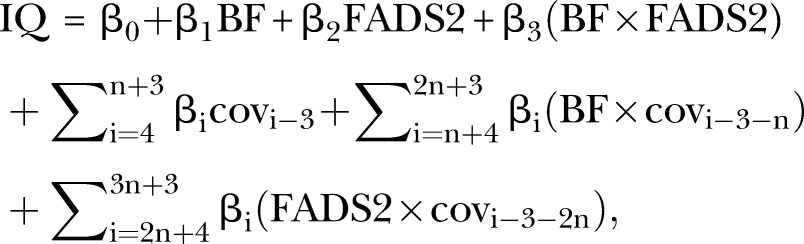
  where:
  BF: breastfeeding (any or exclusive) as a binary, categorical, numerically coded categorical variable or numeric (in months) variable;
  FADS2: *FADS2* polymorphism (rs174575 or rs1535) coded in additive or recessive model;
  cov: generic representation of a covariate;
  n: number of covariates included in the analysis.
  Given that all analysis will be performed three times (unadjusted and two adjusted models), up to 240 regression analyses will be performed. For studies that meet the minimal eligibility criteria, this number will be 12. The potential confounding effect of covariates on the interaction between breastfeeding and *FADS2* will be properly modelled by including interaction terms of breastfeeding and *FADS2* polymorphism with each covariate.^36^
3. The primary analysis will use any breastfeeding in binary form and a recessive genetic model in unadjusted and adjusted models. This corresponds to a replication of the main analysis performed by Caspi *et al*^18^ and Steer *et al*^20^ The remaining analyses are aimed at further exploring the *FADS2*-breastfeeding interaction by evaluating different genetic models, and whether or not there are dose-response breastfeeding effects. Covariate adjustment; regarding covariate adjustment, three analyses will be performed:
  - Unadjusted (model 1);
  - Adjusted for sex, age and age^2^. Multicentric studies or studies with genome-wide genotyping data available will also control for field centre or ancestry-informative principal components, respectively (model 2);
  - Adjust for the same covariates listed above, and also for maternal education and (maternal education)², and/or maternal cognition and (maternal cognition)^2^ (model 3).
4. *Meta-analysis*: Descriptive statistics will be checked for potential errors which will be corrected before conducting the meta-analysis. We will then conduct a preliminary analysis to evaluate if there is heterogeneity due to a few studies; if so, the coordinating team will contact these studies individually for identification of potential errors or problems. In case no issues are identified, the study(ies) will be included in the meta-analysis.
  After checking for these potential sources of artificial heterogeneity, we will then conduct the final meta-analysis. We will report both fixed-effects and random-effects, and use meta-regression to evaluate the following sources of heterogeneity: age, prevalence and duration of breastfeeding, retrospective versus prospective breastfeeding information, measures of IQ, adjustment for principal components and continental region. The main statistics that we will report are the pooled linear regression coefficients for breastfeeding (corresponding to the effect among individuals in the baseline *FADS2* genotype), *FADS2* (corresponding to the effect among individuals never breastfed) and *FADS2*-breastfeeding interaction. We will also report heterogeneity statistics and subgroup-specific estimates, as well as descriptive statistics from each contributing study.
5. *Sensitivity analysis*: We will compare overall meta-analytical estimates with results obtained using subsets of all studies. In case heterogeneity is detected, we will also report estimates for homogeneous subgroups in order to understand if some sources of heterogeneity could be attributed to bias. For example, subsetting based on sample size or length of recall of information on breastfeeding duration may yield insights on the influence of publication or recall bias (respectively) in the estimates.
  To explore the possibility of bias due to gene–environment correlation, we will repeat *FADS2-*breastfeeding interaction analysis having maternal education (converted to US years of education based on ISCED standards, as reported previously^37^) and maternal cognition as the outcome variable. Since only models 1 and 2 will be performed for these outcomes, there will be 160 regression analyses for each. Added to the 240 analyses for IQ, de novo results from 560 regression analyses (performed automatically by the scripts provided) will be obtained from studies that contribute to all analyses.

### Sample size calculation

Sample size requirements to detect a *FADS2*-breastfeeding interaction were evaluated through simulations (5000 simulations per combination of parameters) using R V.3.2.4.

The following parameters were evaluated:

- Prevalence of ever being breastfed: 85% and 95%. These values are based on the estimates recently provided by Victora *et al*^4^ for high-income countries and for countries in all other income groups, respectively.
- Prevalence of the GG genotype: 7.2% and 12.9%. These values were obtained from the 1000 Genomes (phase 3) Project Browser for the rs174575 and rs1535 SNPs (respectively) in European populations.
- Mean difference in IQ according to *FADS2* polymorphism among individuals never breastfed, comparing GG individuals with non-G carriers: −2.15, −4.3 and −8.6. The intermediate value (−4.3) corresponds to the results from Steer *et al*^20^ which is the largest study that evaluated the *FADS2*-breastfeeding interaction on IQ to date. The remaining values correspond to half and twice the effect reported by Steer *et al*, and were used to evaluate sample size requirements in case of weaker and stronger *FADS2* effects.
- Mean difference in IQ according to *FADS2* polymorphism among ever breastfed individuals: zero and half the effect in the never breastfed group. Lack of *FADS2* effect among ever breastfed individuals correspond to the DHA hypothesis described above.
- Sample size (10 000, 12 500, 15 000, 17 500 and 20 000 individuals).

All possible combinations of the above parameters correspond to 120 simulation scenarios. In all of them, the outcome variable was normally distributed (mean=100 and SD=10) and *FADS2* and breastfeeding were independent. p Values for the interaction coefficient were obtained from linear regression models (two-sided t tests). Power was defined as the proportion of tests with p<0.05.

Among the 120 simulation scenarios, power was <80% in only seven of them. The most critical scenario was when breastfeeding prevalence was 95%, GG prevalence was 7.2%, *FADS2* effect among individuals never breastfed was −2.15, and the effect among ever breastfed individuals was half the latter (power=77.3% for a sample size of 20 000 individuals). When sample size was up to 12 500 individuals, power was also <80% when GG prevalence was 12.9%.

It is important to consider that none of the scenarios were underpowered when breastfeeding prevalence was 85%. This estimate is likely to apply to this study better than the value of 95% given that our focus is on individuals of European ancestry (therefore, samples from high-income countries are more likely to be eligible). Moreover, none of the scenarios was underpowered when *FADS2* effect was at least equal to the effect reported by Steer *et al* (which is the best estimate currently available), as well as when there was no *FADS2* effect among ever breastfed individuals.

Therefore, in the majority of realistic scenarios, a sample size of 10 000 individuals would allow properly powered primary analysis. On the basis of a preliminary identification of eligible studies, achieving such sample size is feasible.

### Ethics statement

Only studies with appropriate ethical approval will be considered to participate. Only summary-level statistics (rather than individual-level data) will be shared between the individual study and the coordinating team. Therefore, the present study does not require additional ethical approval other than what has already been provided to participating studies individually. We will obtain all necessary institutional approvals to conduct the analysis.

## Discussion

This collaborative meta-analysis has the potential to improve the understanding of the effect modification of *FADS2* variants on the association between breastfeeding and IQ. However, the study has some limitations.

To achieve a larger sample size and allow participation of different studies, some compromises are necessary. Particularly, we will include breastfeeding measures with different recall times, as well as IQ measures that differ regarding test, subtests included and/or age at measurement. Although a large sample size will contribute to minimise limitations due to heterogeneity (which will also be evaluated in detail), such inconsistencies might still influence the results.

Second, the analysis will be limited to singletons of European ancestry. This will likely reduce heterogeneity (eg, due to systematic differences in breastfeeding patterns comparing twins with singletons), and bias (eg, due to population stratification). Moreover, most genetic epidemiology studies to date have been conducted in Europeans, so it is unlikely that restricting this to Europeans will incur substantial sample size losses. However, it may limit the external validity of our findings.

Third, several heterogeneity tests will be performed. However, it is difficult to identify all potential sources of heterogeneity. Moreover, it may occur that, in some cases, subsetting studies based on heterogeneity-associated factors result in small subgroups, thus yielding imprecise subgroup-specific estimates.

Fourth, availability of maternal education or maternal cognition measures was not included as one eligibility criterion. Although we recognise the importance of accounting for these variables in studies involving breastfeeding and IQ, we opted by allowing studies without these data to participate for two main reasons. First, it is likely that requiring these data would substantially reduce the sample size. Second, previous publications observed no major implication of such measures on *FADS2*-breastfeeding interaction.^18^ ^20^ Therefore, we opted for an inclusive approach coupled with sensitivity analyses using the subset of studies with these data.

Finally, based on sample size calculations under a variety of realistic situations, we expect to have enough power to detect interaction effects. However, a lack of strong statistical association could be a result of small effects and/or heterogeneity that we fail to account for. Moreover, given the inconsistencies among published studies and the fact that we will properly control for confounding in the interaction setting, it is also possible that our meta-analysis suggests that there is no *FADS2*-breastfeeding interaction (although such strong conclusion might not be feasible due to sample size limitations).

Understanding the health effects—and associated mechanisms—of breastfeeding is important to obtain a more accurate view of the impact of breastfeeding promotion. This, in turn, may have implications regarding the extent to which investments on such promotion should be prioritised over other public health initiatives. Identifying the mechanisms could also be important to incorporate key nutritional components of breast milk into formula milk.

Regarding effect modification (if any) of *FADS2* variants on the association between breastfeeding and IQ, individual studies published to date are inconsistent. Improving current understanding of this interaction might yield biological insights regarding the importance of LC-PUFAs for breastfeeding effects. This research question will be addressed using a collaborative meta-analysis based on consistent a priori defined analysis of harmonised data. Therefore, publishing this protocol will reduce potential biases associated with data mining, thus contributing to generation of reliable evidence.
