# Correction: *Effect modification of FADS2 polymorphisms on the association between breastfeeding and intelligence: protocol for a collaborative meta-analysis*

**DOI:** 10.1136/bmjopen-2015-010067corr1

**Published:** 2016-08-03

Hartwig FP, Davies NM, Horta BL, *et al.* Effect modification of FADS2 polymorphisms on the association between breastfeeding and intelligence: protocol for a collaborative meta-analysis. *BMJ Open* 2016;6:e010067. This paper has been resupplied with the correct license statement.

